# Lycopene alleviates oxidative stress via the PI3K/Akt/Nrf2pathway in a cell model of Alzheimer’s disease

**DOI:** 10.7717/peerj.9308

**Published:** 2020-06-08

**Authors:** Yinchao Fang, Shanshan Ou, Tong Wu, Lingqi Zhou, Hai Tang, Mei Jiang, Jie Xu, Kaihua Guo

**Affiliations:** 1 Department of Anatomy and Neurobiology, Zhongshan School of Medicine, Sun Yat-sen University, Guangzhou, China; 2 The 5th Affiliated Hospital, Sun Yat-sen University, Zhuhai, China; 3 Guangdong Jiangmen Chinese Medical College, Jiangmen, China; 4 Guangdong Province Key Laboratory of Brain Function and Disease, Zhongshan School of Medicine, Sun Yat-sen University, Guangzhou, China

**Keywords:** Lycopene, M146L cell, Oxidative stress, Apoptosis

## Abstract

**Background & Aims:**

Oxidative stress (OS) plays an important role in neurodegenerative diseases such as Alzheimer’s disease (AD). Lycopene is a pigment with potent antioxidant and anti-tumor effects. However, its potential role in central nervous system is not well-defined. The aim of this study was to investigate the effect of lycopene on the cell model of AD and determine its underlying mechanisms.

**Methods:**

M146L cell is a double-transfected (human APP gene and presenlin-1 gene) Chinese hamster ovary (CHO) cell line that overexpresses β -amyloid (Aβ) and is an ideal cell model for AD. We treated cells with lycopene, and observed the effect of lycopene on M146L cells.

**Results:**

Oxidative stress and apoptosis in M146L cells were significantly higher than those in CHO cells, suggesting that Aβ induced OS and apoptosis. Lycopene alleviated OS and apoptosis, activated the PI3K/Akt/Nrf2 signaling pathway, upregulated antioxidant and antiapoptotic proteins and downregulated proapoptotic proteins. Additionally, lycopene inhibited β -secretase (BACE) activity in M146L cells. These results suggest that lycopene inhibits BACE activity and protects M146L cells from oxidative stress and apoptosis by activating the PI3K/Akt/Nrf2 pathway.

**Conclusion:**

Lycopene possibly prevents Aβ-induced damage by activating the PI3K/Akt/Nrf2 signaling pathway and reducing the expression of BACE in M146L cells.

## Introduction

Alzheimer’s disease (AD) is a neurodegenerative disease with an insidious onset and slow progression of memory impairment, cognitive impairment and decreased executive ability. It is pathologically characterized by the formation of senile plaques and neurofibrillary tangles. Normally, amyloid precursor protein (APP) is first cleaved by α-secretase to produce soluble APP (sAPP), which is associated with signal transduction and participates in synaptic plasticity, learning and memory, emotional behavior, and nerve survival. The presenilin (PS) exert a crucial role in the pathogenesis of AD by mediating the intramembranous cleavage of APP (Oikawa & Walter, 2019). PS1 is the core hydrolytic component of γ-secretase (Steiner, Fluhrer & Haass, 2008). APP is successively cleaved by β-secretase and γ-secretase producing Aβ and forming plaques under pathological conditions. Accumulation of A β leads to blockage of ion channels, imbalances in calcium homeostasis, mitochondrial oxidative stress, impaired energy metabolism, and abnormal sugar regulation, ultimately leading to nerve cell death (Vassar et al., 1999; Wang et al., 2017). M146L, which has been transfected with human APP gene and PS1 gene and expresses Aβ consistently and steadily, is an ideal cell model for AD research.

Oxidative stress refers to the imbalance between oxidation and antioxidation in the body with excessive free radical production. Physiological homeostasis of oxidative stress is crucial for the maintenance of oxidative signal transduction, however excessive oxidative stress breaks the balance and causes damage. OS is a negative effect produced by free radicals in the body and is an important factor leading to aging and diseases, as well as apoptosis. OS is closely related to aging and chronic diseases and has a pivotal role in the neurodegenerative process through different pathways (Tonnies & Trushina, 2017). Apoptosis triggered by OS results in demyelination of neurons, and dysfunction of proteasomes caused by OS induces accumulation of oxidized proteins in the cytoplasm, formation of senile plaques, neurodegeneration and neuronal death (Yaribeygi et al., 2018).

The phosphatidyl inositol 3-kinase (PI3K)/ protein kinase B (Akt) signaling pathway is widely involved in the regulation of cell metabolism, survival and apoptosis and is related to the occurrence and development of AD (Zaplatic et al., 2019). Nuclear factor erythroid 2-related factor 2 (Nrf2) is a transcription factor that is directly regulated by glycogen synthase kinase 3β (GSK3β) in the PI3K/Akt pathway (Ali et al., 2018). Nrf2 induces antioxidants and detoxication, such as glutamate cysteine ligase catalytic subunit (Gclc) and glutamate cysteine ligase modifier subunit (Gclm) (Paladino et al., 2018). It has been reported that the Nrf2 pathway is a target for the treatment of neurodegenerative diseases (Bahn & Jo, 2019; Esteras, Dinkova-Kostova & Abramov, 2016). The absence of Nrf2 is associated with increased amyloidopathy and exacerbates cognitive deficits, which are associated with the early onset of AD (Rojo et al., 2017).

Lycopene, a red carotenoid found in a variety of vegetables and fruits, is a natural antioxidant. It is a well-known fat- soluble carotenoid, and has been studied for the treatment of tumors (Chen et al., 2015), cardiovascular diseases (Cheng et al., 2017) and even neurodegenerative diseases (Kumar & Kumar, 2009; Liu et al., 2013), and shows significant antioxidant and antiapoptotic effects (Tang et al., 2008; Lin et al., 2018). Lycopene has also been reported to reduce damage caused by Aβ (Wang et al., 2018; Qu et al., 2016). Some recent reports show that lycopene can improve cognitive function (Crowe-White, Phillips & Ellis, 2019; Wang et al., 2019). In this study, M146L cells were used to verify our previous results and further evaluate the role of lycopene in alleviating oxidative stress and reducing apoptosis and its mechanism in vitro. Verification of the underlying mechanism of the antioxidant and antiapoptotic effects of lycopene, and characterization of the effects induce by lycopene in M146L as model of AD.

## Material and Methods

### Cell cultures and treatments

CHO cells were obtained from Conservation Genetics of the Chinese Academy of Sciences Kunming Cell Bank, and M146L cells were purchased from Bailey Biological Technology Company, Shanghai. The cells were cultured in high-glucose Dulbecco’s modified Eagle’s medium (ThermoFisher Scientific, USA) supplemented with 10% fetal bovine serum (ThermoFisher Scientific, USA) and 1% penicillin/streptomycin solution (ThermoFisher Scientific, USA) at 37 °C and 5% CO_2_. G418 (400 µg/ml, Sigma-Aldrich, USA) was used for the generation of stable M146L cell lines.

Lycopene (Sigma-Aldrich, MO, USA) was solubilized in tetrahydrofuran containing 0.025% butylated hydroxytoluene (Sigma-Aldrich, MO, USA). Lycopene was added to the cells at a concentration of 10 µM for 24 h. For the inhibitor study, M146L cells were pretreated with LY294002, a sp(APExBIO, USA) at 10 µM for 1 h before treatment with lycopene.

### Assay of oxidative stress

The reactive oxygen species (ROS) assay was performed using a ROS Assay Kit (Beyotime, China) according to the manufacturer’s protocol. Malondialdehyde (MDA) was assayed using a MDA Assay Kit (Beyotime, China) according to the manufacturer’s procedure.

### Western blot assays

Proteins were prepared using a protein extraction kit (BestBio, China) according to the manufacturer’s instructions. The protein concentration was determined using a BCA kit (Beyotime, Beijing, China) and the samples were then boiled for 5 min in sodium dodecyl sulfate (SDS) loading buffer to denature the proteins. Equal amounts of protein from each sample were separated by SDS-PAGE and transferred to poly vinylidene fluoride (PVDF) membranes. The membrane was blocked with 5% bovine serum albumin in Tris-Buffered Saline and Tween 20 (TBST) for 1 h at room temperature, and the separated proteins were incubated overnight at 4 °C with primary antibodies for the target proteins β-actin (1:5000, Proteintech, USA), glyceraldehyde-3phosphate dehydrogenase (GAPDH) (1:5000, Proteintech, USA), Nrf2 (1:1000, CST, USA), Gclc (1;1000, Abcam, USA), Gclm (1:1000, Abcam, USA), Akt (1:1000, CST, USA), p-Akt-Ser473 (1:1000, CST, USA), GSK3β (1:1000, CST, USA), p-GSK3β-Ser9(1:1000, CST, USA), Bcl-2 (1:1000, Abcam, USA), activated- caspase-3 (1:200, Abcam, USA), BACE (1:1000, CST, USA), and APP (1:1000, CST, USA). Following incubation with species-specific horseradish peroxidase (HRP)-conjugated secondary antibody at room temperature for 1 h, the blots were developed using a chemiluminescence substrate. The corresponding bands were detected using a GE AI600 Imaging System (GE, USA), and the band densities were quantified using Image J software and normalized to β-actin or GAPDH.

### Annexin V and PI staining

The apoptotic rate in M146L cells was detected using an Annexin V-FITC apoptosis detection kit (BestBio, China). The cells were collected and re- suspended in 400 µL Annexin V binding buffer and then stained with 5 µL Annexin V-FITC for 15 min at 4 °C in the dark. Finally, the cells were stained with 10 µl of propidium iodide (PI) for 5 min at 4 °C in the dark and immediately analyzed by flow cytometry using a CytoFLEX Detection System (Beckman Coulter, Germany).

### Statistical analysis

Statistical analysis was performed using SPSS 22.0. Data are presented as the mean ± SD of at least three independent experiments. Analysis was performed using one- way analysis for post hoc test, and *P* < 0.05 was considered statistically significant.

## Results

### Lycopene prevents oxidative stress in M146L cells

**Figure 1:**
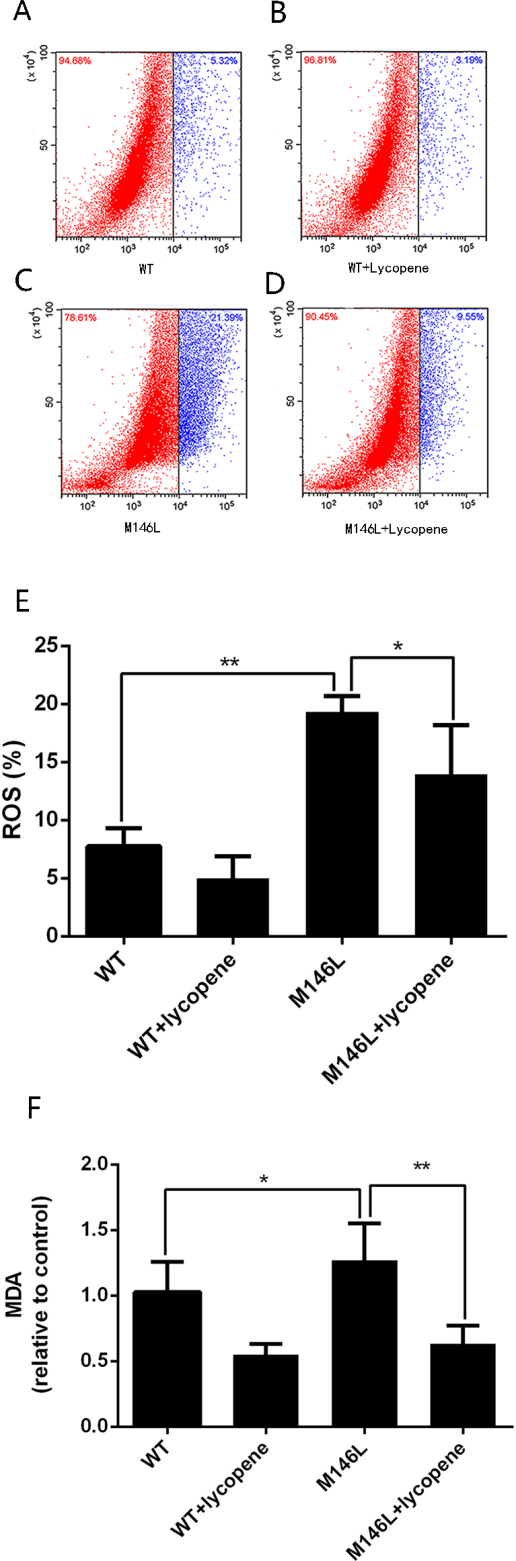
Lycopeneprotects M146L cells from oxidative stress. (A–D) Intracellular ROS was measured by flow cytometry analysis using DCFH-DA, (E) quantitative analysis showing the ROS ratio. (F) MDA was assessed by using the Lipid Peroxidation MDA Assay Kit. Data are expressed as means ±SD; WT: CHO cells; ^∗^*p* < 0.05, ^∗∗^*p* < 0.01, compared with the M146L group.

We analyzed ROS and MDA in M146L and WT cells with or without lycopene treatment. As shown in Fig. 1A and 1B, the expression of ROS in M146L cells was much higher than that in WT cells, and after treatment with lycopene, ROS were reduced in both M146L and WT cells. A similar pattern was observed regarding MDA (Fig. 1C). These results suggest that Aβ induces oxidative stress and that lycopene prevents stress.

### Lycopene increases the antioxidant enzymes Gclc and Gclm in M146L cells

**Figure 2:**
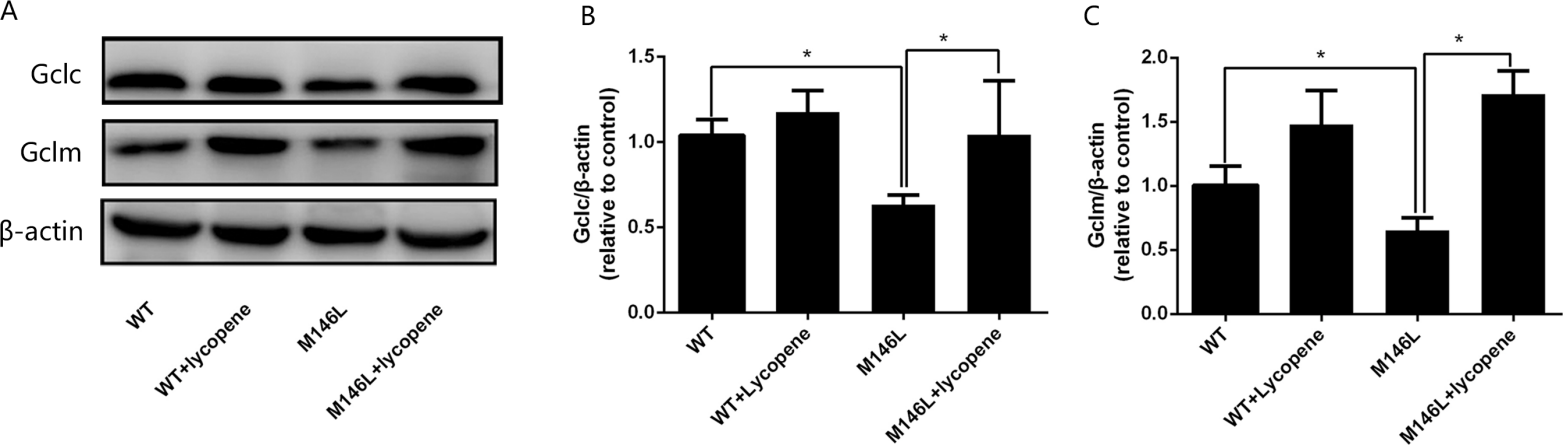
Lycopeneup-regulating the levels of Gclc and Gclm. (A) The expression of Gclc and Gclm were detected by Western blot. (B-C) Densitometric analysis of the proteins normalized to β-actin. Date were expressed as means ±SD; WT: CHO cells; ^∗^*p* < 0.05, ^∗∗^*p* < 0.01, compared with the M146L group.

Western blotting was used to detect the expression of proteins (Fig. 2A). As demonstrated in Figs. 2B and 2C, the expression of Gclc and Gclm in M146L cells was lower than that in WT cells, suggesting that Aβ inhibits the expression of antioxidant enzymes. Lycopene promoted their expression. The results suggest that lycopene has an antioxidant effect.

### Lycopene activates the PI3K/Akt/Nrf2 pathway in M146L cells

**Figure 3:**
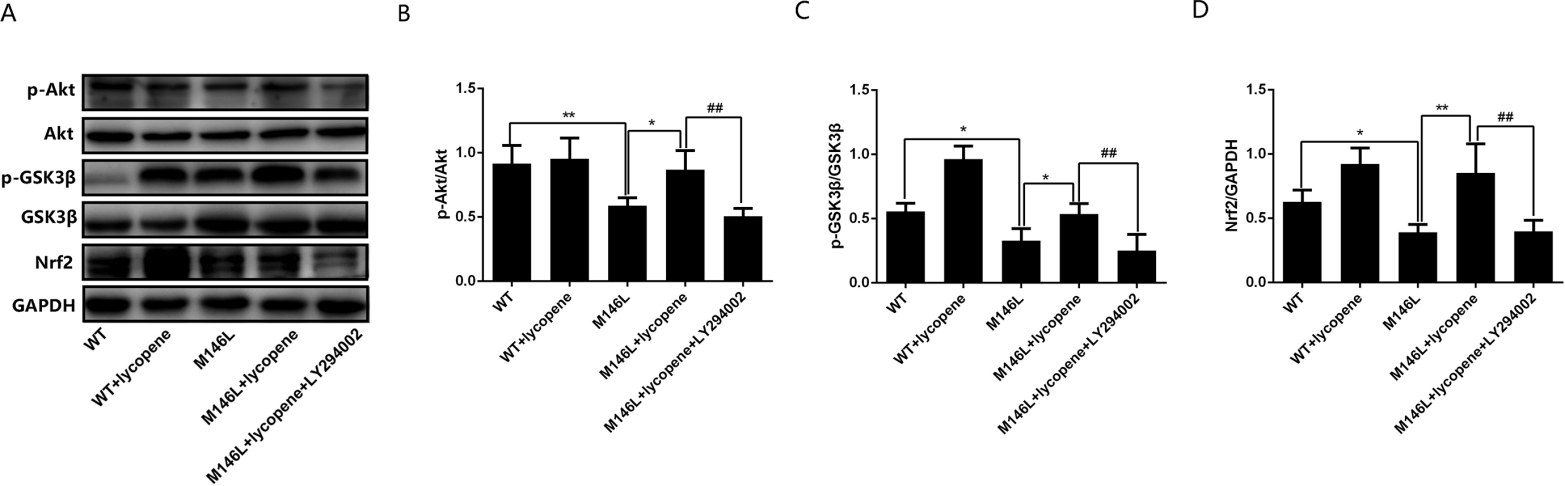
Lycopene activated the PI3K/Akt pathway. (A) The expression of protein was detected by Western blot. (B) The protein level of Akt and p-Akt were detected by Western blot and the relative optical density. (C) The protein level of GSK3 β and p-GSK3β were detected by Western blot and the relative optical density. (D) The protein level of Nrf2 and densitometric analysis normalized to GAPDH. Data are expressed as means ± SEM; WT: CHO cells; ^∗^*p* < 0.05, ^∗∗^*p* < 0.01, compared with the M146L group; ^#^*p* < 0.05, ^##^*p* < 0.01, compared with M146L+Lycopene group.

Western blotting was used to detect the expression of proteins (Fig. 3A). As shown in Figs. 3B and 3C, the phosphorylation of Akt and GSK3β in M146L cells was decreased compared with WT group. These results indicate that Aβ inhibits the activation of this pathway. Lycopene induced the phosphorylation of Akt and GSK-3 β, and the effects were blocked when the cells were pretreated with LY294002. A similar pattern had observed for Nrf2 (Fig. 3D). These results suggest that lycopene plays an oxidative stress role by activating the PI3K/Akt/Nrf2 pathway.

### Lycopene alleviates apoptosis in M146L cells

**Figure 4:**
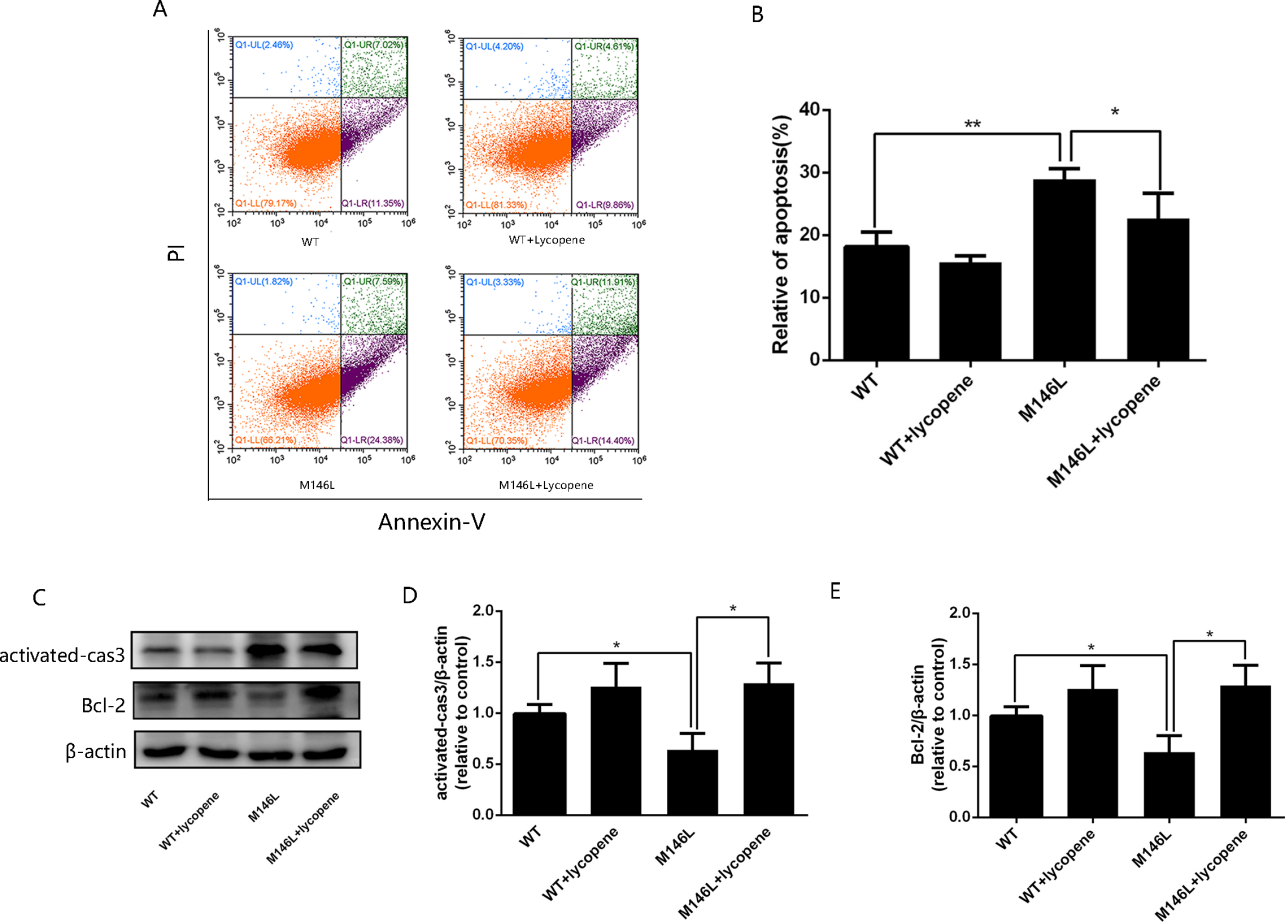
Lycopenealleviated apoptosis in M146L cells. (A) Flow cytometry plots showing. Early apoptotic cells (Annexin V+/PI − ) are in quadrant Q1-LR; late apoptotic cells (Annexin V+/PI+) are in quadrant Q1-UR; normal cells (Annexin V − /PI − ) are in quadrant Q1-LL; and late necrotic cells injured by experimental manipulation (Annexin V − /PI+) are in quadrant Q1-UL. (B) Quantitative analysis showing the apoptosis ratio. (C) The expression of activated caspase3 and Bcl-2, (D–E) densitometric analysis of the proteins normalized to β-actin. Data are expressed as means ± SD; WT: CHO cells; ^∗^*p* < 0.05, ^∗∗^*p* < 0.01, compared with the M146L group.

Annexin V/PI staining was performed to determine apoptosis (Fig. 4A). The rate of apoptosis in M146L cells was higher than that in WT cells, whereas lycopene decreased the percentage of apoptotic cells (Fig. 4B), suggesting that A β induces apoptosis, while lycopene plays an antiapoptotic role. Expression of activated caspase-3 and Bcl-2 was detected by Western blotting, β-actin in the same sample was detected as the control (Fig. 4C) The relative optical density in shown in Figs. 4D and 4E. As shown in the results, expression of proapoptotic proteins was increased and antiapoptotic proteins were decreased in M146L cells compared to those of WT cells, which was consistent with Aβ-induced apoptosis. Lycopene reduced apoptosis, blocked the expression of proapoptotic proteins, and promoted the expression of antiapoptotic proteins, which is also consistent with the antiapoptotic effect of lycopene.

###  Lycopene inhibits BACE activity in M146L cells

**Figure 5:**
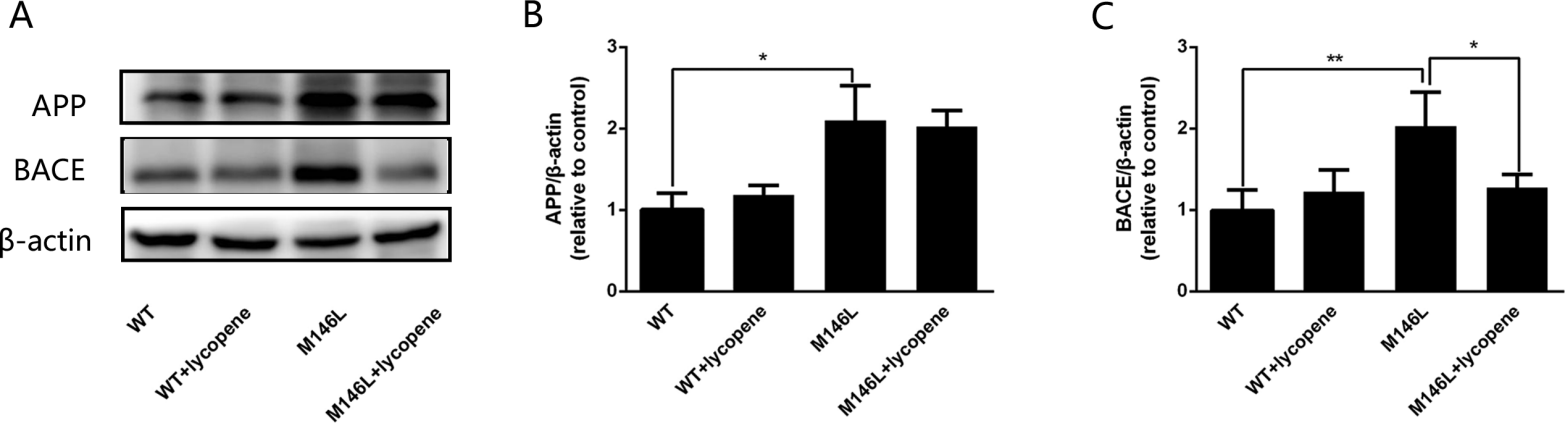
Lycopene inhibits BACE activity in M146L cells. The levels of APP and BACE protein (A). Densitometric analysis of APP normalized to β-actin (B), densitometric analysis of BACE normalized to β-actin (C). Data are expressed as means ± SEM; WT: CHO cells; ^∗^*p* < 0.05, ^∗∗^*p* < 0.01, compared with the M146L group.

Western blotting was used to detect the expression of APP and BACE (Fig. 5A). The level of APP in M146L cells was twice as high as that in WT cells, and the there was an insignificant reduction in these proteins in M146L cells after treatment with lycopene (Fig. 5B). Moreover, the BACE protein level was significantly increased compared with that of the WT group, and lycopene reduced BACE in M146L cells (Fig. 5C). Taken together, these results suggest that lycopene reduces the toxicity of A β by inhibiting BACE activity rather than reducing APP expression.

## Discussion

AD is a progressive neurodegenerative disease and the most common cause of dementia. The formation of senile plaques caused by A β deposition is one of the main pathological features of AD. It is generally accepted that BACE and is a crucial factor in the transformation of APP into Aβ. Studies have reported that increased BACE expression in the brain may be one of the causal factors for AD (Li et al., 2004; Cai et al., 2001). Research reports that aging and chronic diseases are closely related to oxidative stress (Florence, 1995; Wang, Markesbery & Lovell, 2006). Because of its strong antioxidative activity, lycopene has been applied to many oxidative stress- associated diseases. A series of studies suggest that lycopene has preventive and therapeutic effects on cardiovascular diseases, cancer, diabetes, osteoporosis, arthritis, fertility and neurodegenerative diseases (Clinton, 1998; Jain, Agarwal & Rao, 1999). In the present study, we used the M146L cell line, which can stably secrete Aβ, as a model of AD (Huang et al., 2018; Wei et al., 2008). We investigated the effect of lycopene on inhibition of Aβ-induced oxidative stress and apoptosis and the underlying mechanisms, as well as the effect of lycopene on the expression of BACE.

ROS and MDA are biomarkers that are widely used to detect oxidative stress (Sies, Berndt & Jones, 2017). Our results showed that the oxidative stress level of M146L cells was higher than that of WT cells, and this up-regulation was decreased with lycopene treatment, indicating that Aβ increases oxidative stress and that lycopene could significantly alleviates abnormal oxidative stress. Nrf2 is a transcription factor that induces the expression of cytoprotective and antioxidant genes, which are potential targets for the treatment of neurodegenerative diseases (Buendia et al., 2016). Nrf2-related pathways involved in resistance to oxidative stress through the adjustable antioxidants and detoxification genes, such as NAD(P)H: Quinone Oxidoreductase 1 (NQO1) and certain glutathione S-transferases (GSTs) (Huang et al., 2015). The protein expression levels of Nrf2 and its downstream antioxidant proteins in M146L group were lower than those in the WT group, and increased after treatment with lycopene. This indicates that Nrf2 is closely related to Aβ- induced impairment and that lycopene may improve this damage.

PI3K is an important signal transduction molecule in the growth factor superfamily. Once activated with the help of PI3K- dependent kinase (PDK), PI3K activates Akt via phosphorylation of its serine and threonine residues. Then, p-Akt phosphorylates GSK3β, which leads to inactivation of GSK3β. GSK3β is involved in many prevalent disorders, including psychiatric and neurological diseases, inflammatory diseases, and cancer, and regulates the nuclear export and degradation of Nrf2 (Beurel, Grieco & Jope, 2015; Jain & Jaiswal, 2007). p-GSK3β, however, inhibits this action via phosphorylation of Nrf2 and thus inducing its degradation (Rojo, Sagarra & Cuadrado, 2008). As a result, Nrf2 translocate into the nucleus and promotes the transcriptional expression of downstream phase II detoxification genes and exerts antioxidant stress effects (Farr et al., 2014). In t-BHP-induced neuronal damage cell model, lycopene shows the neuroprotective effects of antioxidative damage and antiapoptotic by reducing the phosphorylation of PI3K/Akt, which revealed that protective effects of lycopene is related to activation of the PI3K/Akt pathway (Huang et al., 2019). To confirm that lycopene alleviates oxidative stress via the PI3K/Akt signaling pathway, the PI3K-specific inhibitor LY294002 was used (Cui, Leng & Wang, 2019; Liu et al., 2019). Our results showed that the pathway was activated after treatment with lycopene, and the protective effect of lycopene was reversed by treatment with LY294002, suggesting that lycopene may play a role in antioxidant stress by activating Nrf2 via the PI3K/ Akt signaling pathway.

Apoptosis refers to programmed cell death, which is an activated process related to the expression and regulation of a series of related genes. OS is associated with apoptosis (Zhao et al., 2013). The B-cell lymphoma-2 (Bcl-2) family and caspases play an important role in regulating apoptosis. As an antiapoptotic protein, Bcl-2 is regulated by Akt in neuroprotection (Qiu et al., 2016). When apoptosis is initiated, inactive Caspase-3 is cleaved and activated to play a proapoptotic role, while Bcl-2 plays an antiapoptotic role (Jan & Chaudhry, 2019). Some studies indicate that Aβ can induce apoptosis (Xu et al., 2018; Alberdi et al., 2018), and lycopene inhibits Aβ-induced apoptosis (Jeong, Lim & Kim, 2019; Sinwoo Hwang, 2017). We studied the role of lycopene in apoptosis of M146L cells, and the results showed that the apoptotic rate of M146L cells was higher than that in the WT group, and lycopene decreased apoptosis. After treatment with lycopene, expression of the proapoptotic protein activated caspase-3 was decreased, and expression of the apoptotic protein Bcl-2 was increased. These results indicate that lycopene can inhibit Aβ-induced apoptosis.

In AD patients, BACE elevation leads to increased Aβ production and enhanced deposition of amyloid plaques (Li et al., 2004), and it’s probably a potential target for the treatment of AD (Maia & Sousa, 2019). APP is first processed by BACE, which is an indispensable factor in the production of Aβ. A previous research indicated that LY294002 inhibited the decreasing the BACE and PS1, reducing the level of Aβ and improving memory impairment in APP/PS1 transgenic mice (Zhao et al., 2016). Our results showed that the expression of APP and BACE in M146L cells was significantly higher than in WT cells. After treatment with lycopene, there was no significant difference in the expression of APP between the groups, but the BACE expression was significantly decreased. Our data are consistent with previous studies that lycopene reduces the expression of BACE, result in decreasing the level of Aβ by activating PI3K/Akt pathway in AD.

## Conclusion

Aβ increases possibly resulted in excessive oxidative stress and leads to apoptosis. Lycopene possibly prevent Aβ-induced cell damage by activating the PI3K/Akt/Nrf2 signaling pathway and reducing the expression of BACE in M146L cells. Therefore, lycopene may have potential in the treatment of AD.

##  Supplemental Information

10.7717/peerj.9308/supp-1 Supplemental Information 1 Experimental data and statistical analysis

## Additional Information and Declarations

### Data Availability

The following information was supplied regarding data availability: The raw measurements are available in the Supplementary Files.
