# Supplementary figures and images for: Lycopene alleviates oxidative stress via the PI3K/Akt/Nrf2pathway in a cell model of Alzheimer’s disease
Source: PeerJ. 2020 Jun 8;8:e9308. doi: 10.7717/peerj.9308 (PMC7289143; doi:10.7717/peerj.9308)

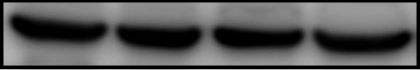

Supplement: Supplemental Information 1 [file peerj-08-9308-s001.zip › Raw date/actin-1 2019.04.29.jpg]

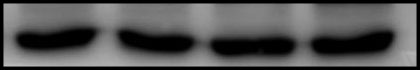

Supplement: Supplemental Information 1 [file peerj-08-9308-s001.zip › Raw date/actin-2.jpg]

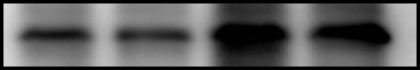

Supplement: Supplemental Information 1 [file peerj-08-9308-s001.zip › Raw date/activated-cas3.jpg]

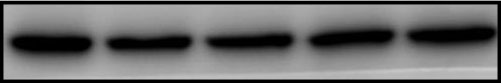

Supplement: Supplemental Information 1 [file peerj-08-9308-s001.zip › Raw date/Akt 2019.06.15.jpg]

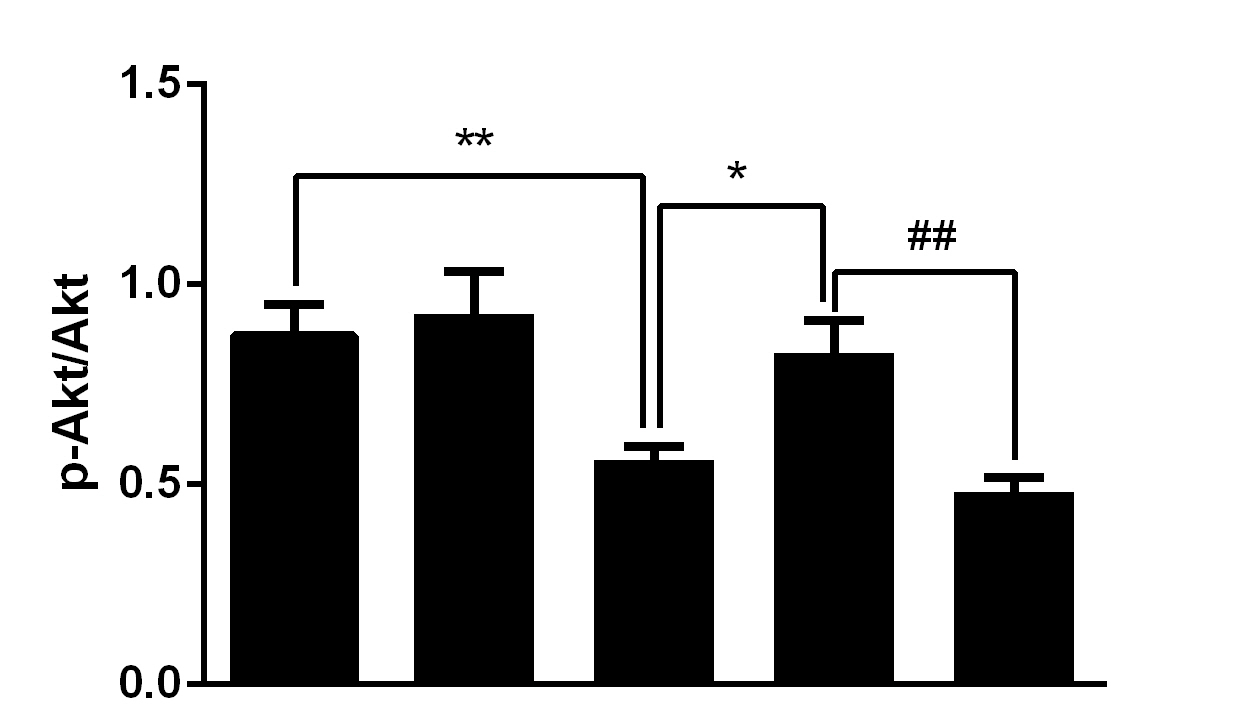

Supplement: Supplemental Information 1 [file peerj-08-9308-s001.zip › Raw date/AKT.jpg]

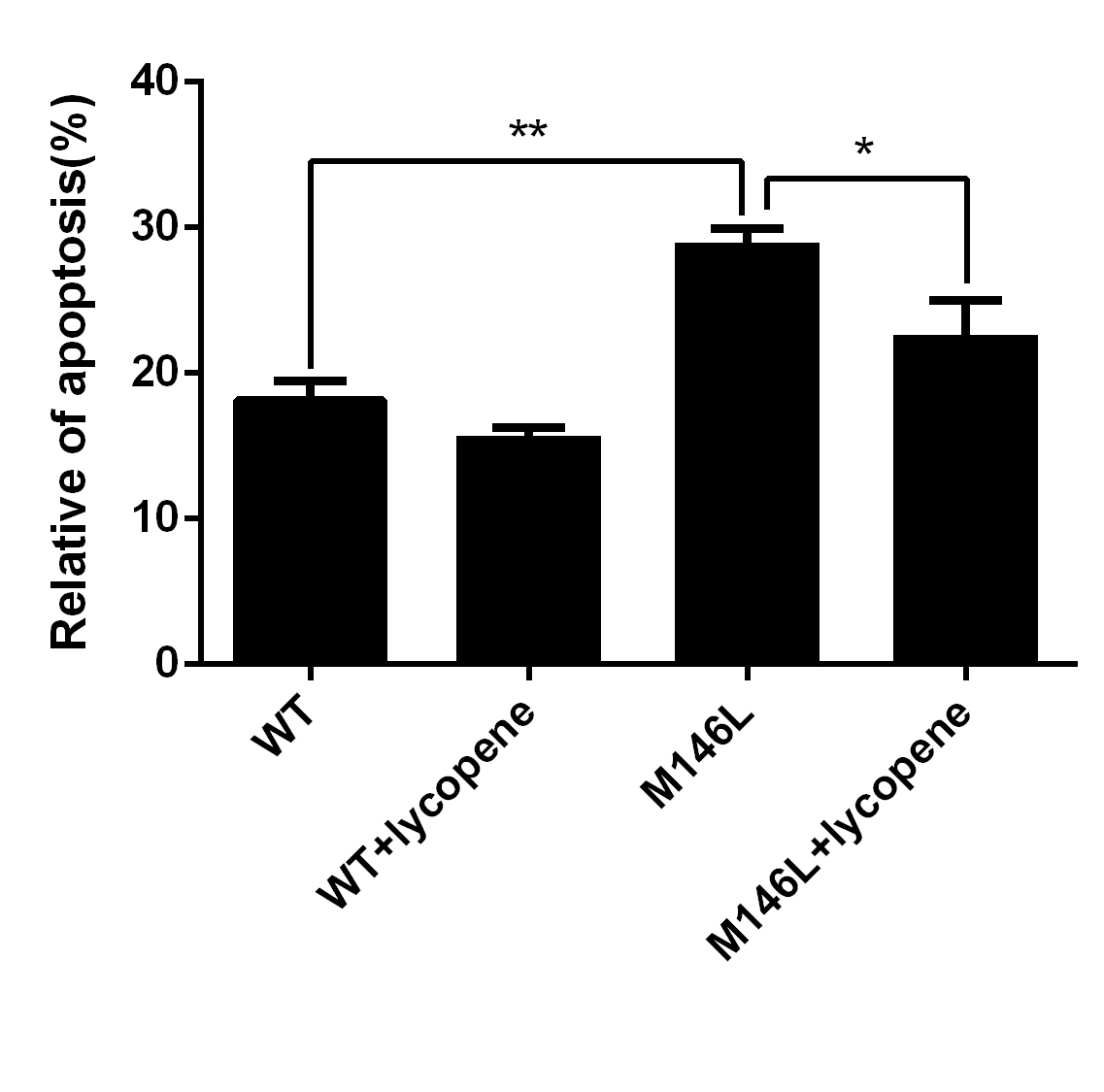

Supplement: Supplemental Information 1 [file peerj-08-9308-s001.zip › Raw date/Apoptosis.jpg]

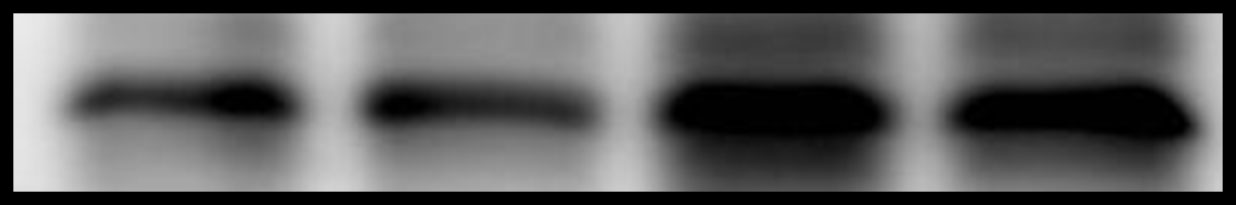

Supplement: Supplemental Information 1 [file peerj-08-9308-s001.zip › Raw date/APP-1.jpg]

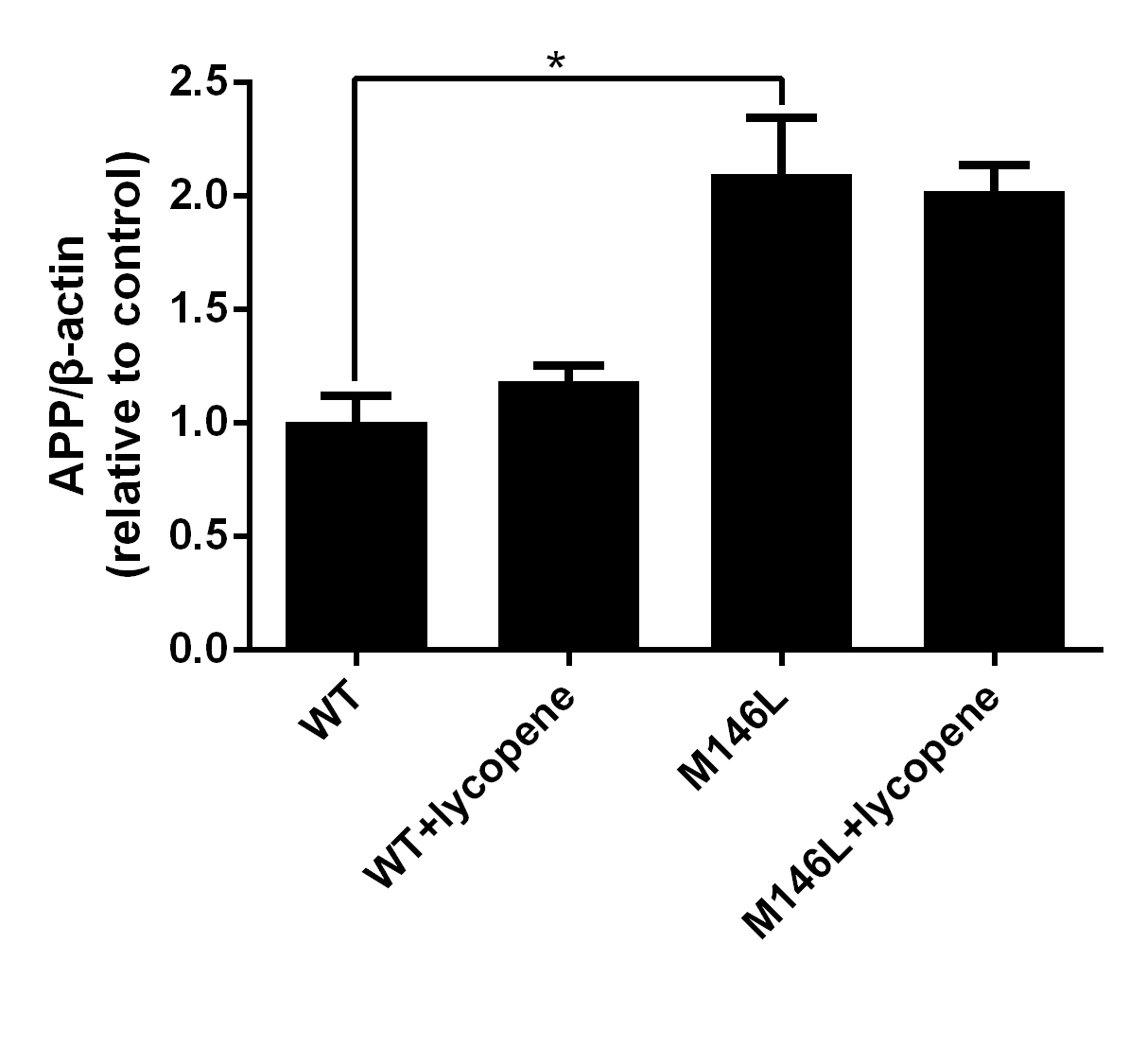

Supplement: Supplemental Information 1 [file peerj-08-9308-s001.zip › Raw date/APP.jpg]

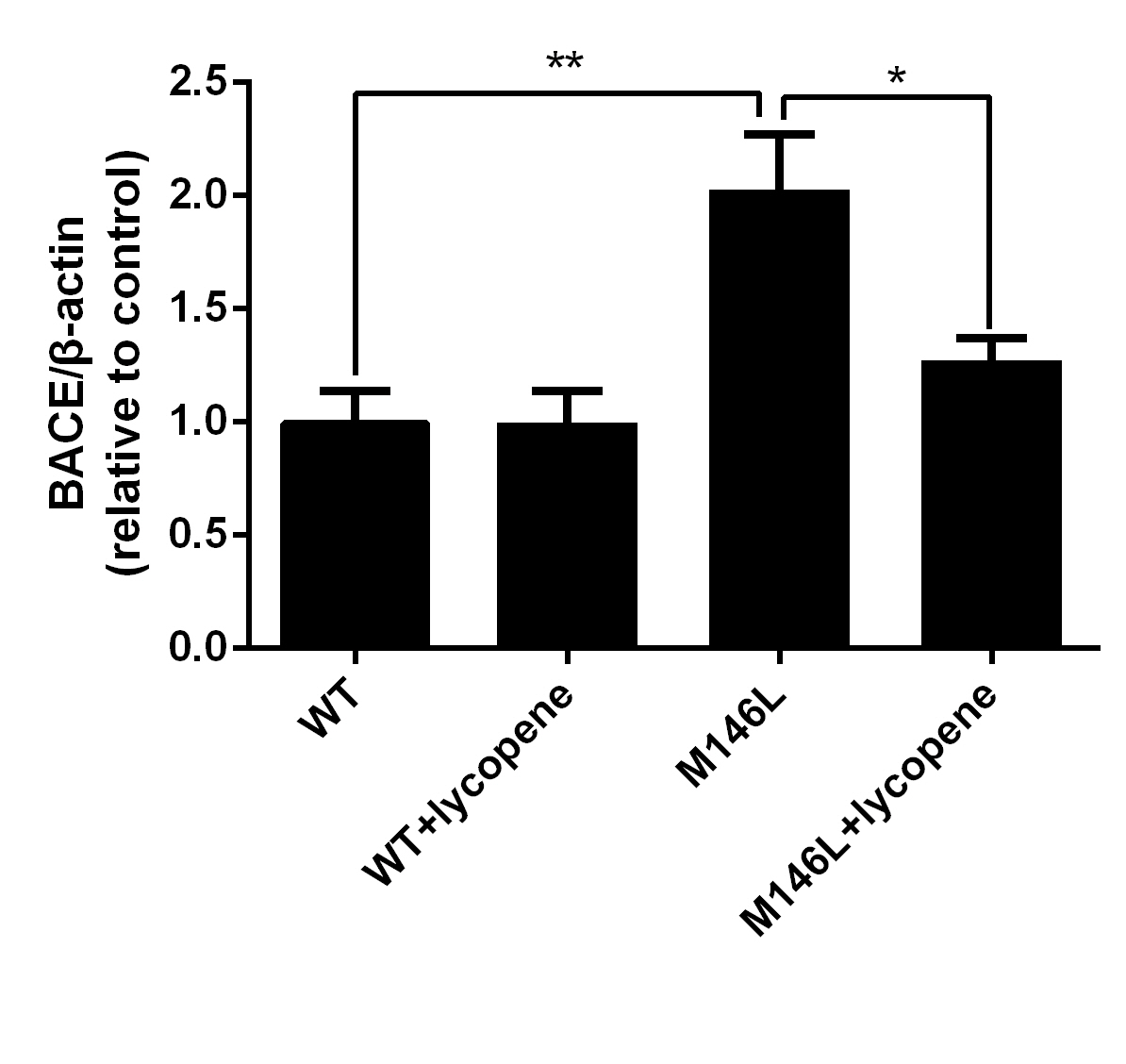

Supplement: Supplemental Information 1 [file peerj-08-9308-s001.zip › Raw date/BACE.jpg]

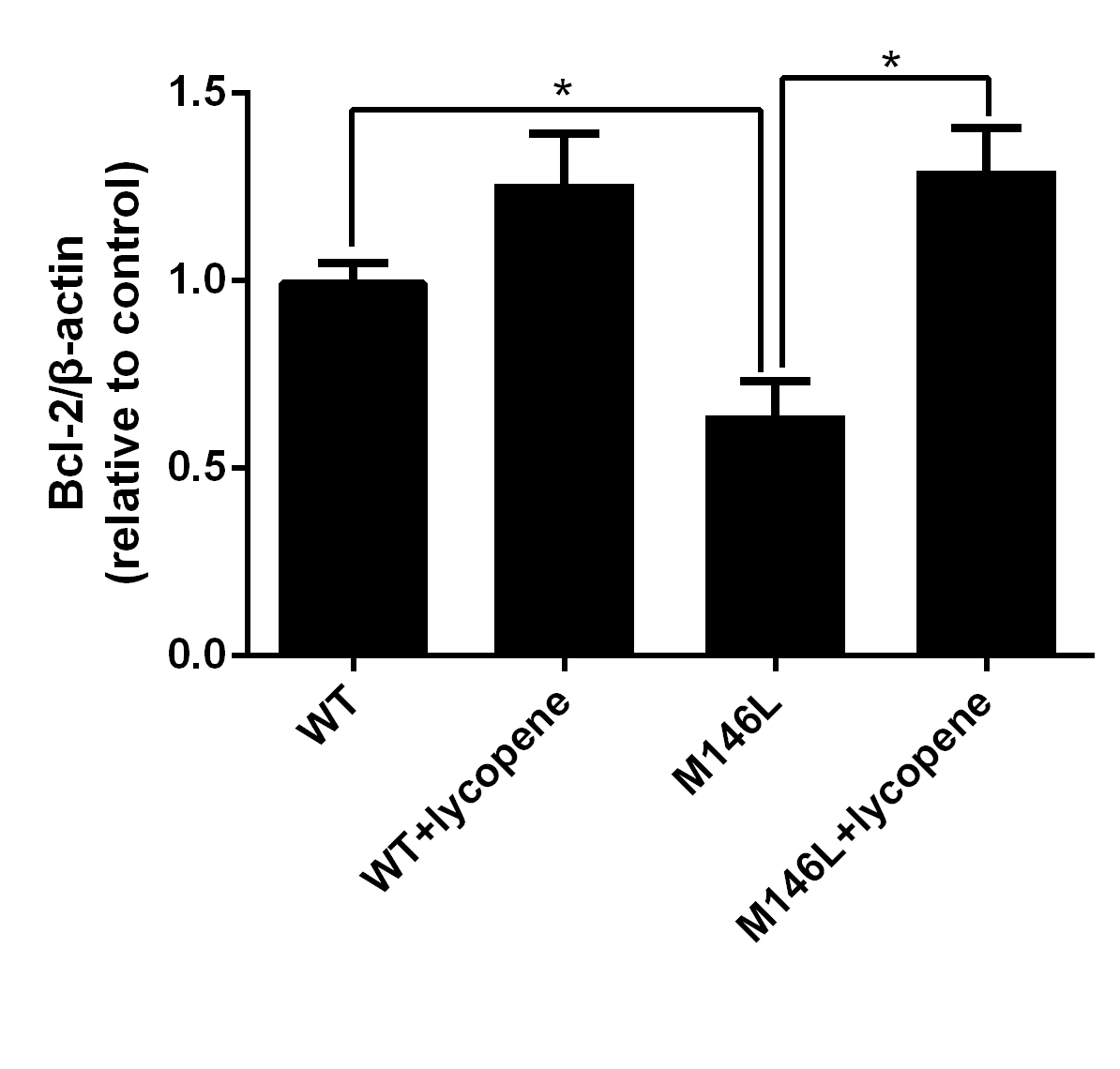

Supplement: Supplemental Information 1 [file peerj-08-9308-s001.zip › Raw date/bcl2-2.jpg]

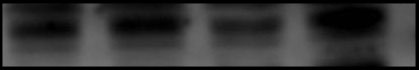

Supplement: Supplemental Information 1 [file peerj-08-9308-s001.zip › Raw date/bcl2.jpg]

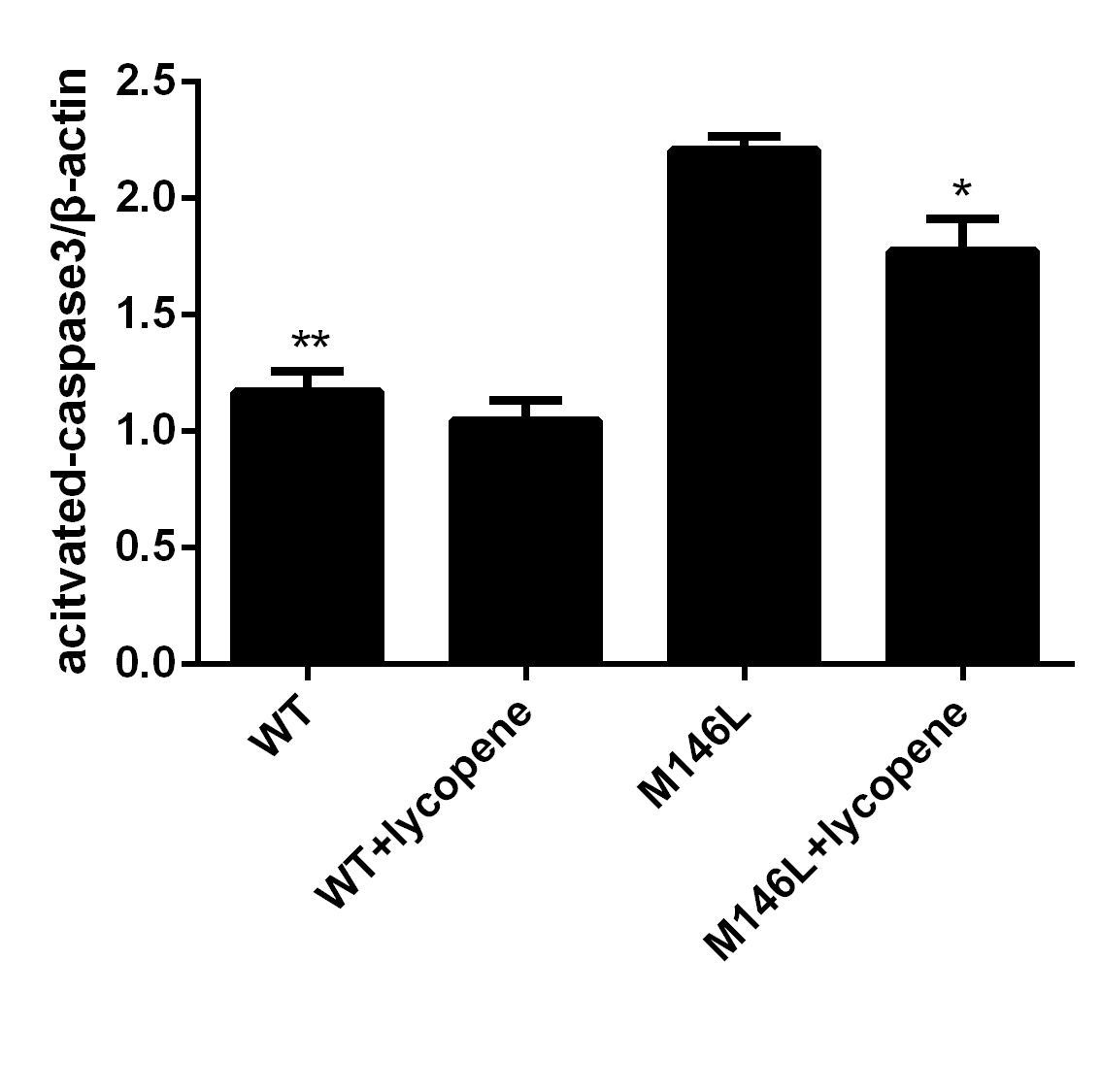

Supplement: Supplemental Information 1 [file peerj-08-9308-s001.zip › Raw date/casp.jpg]

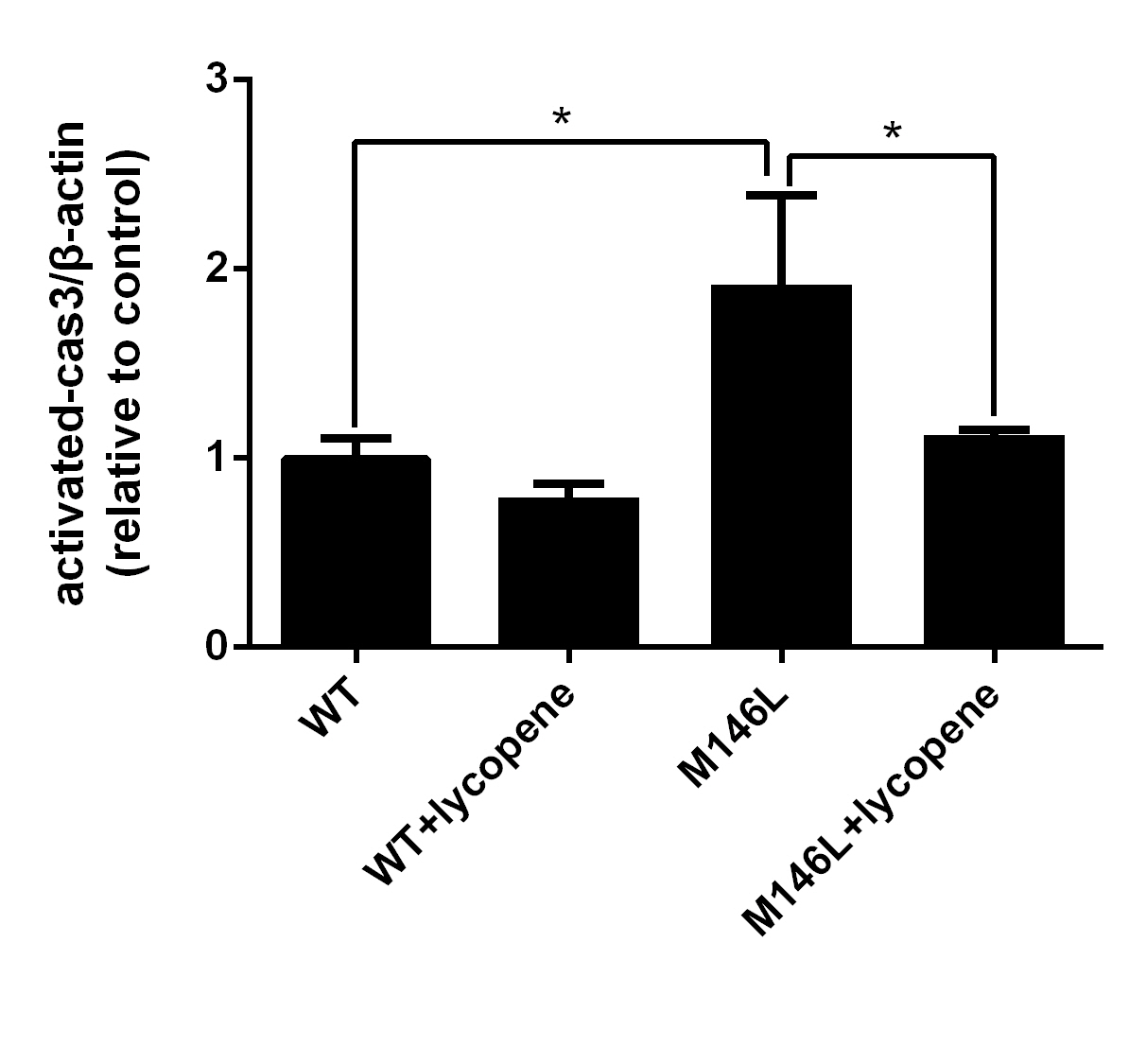

Supplement: Supplemental Information 1 [file peerj-08-9308-s001.zip › Raw date/Data 1.jpg]

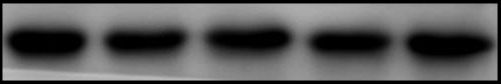

Supplement: Supplemental Information 1 [file peerj-08-9308-s001.zip › Raw date/gapdh2019.06.1.jpg]

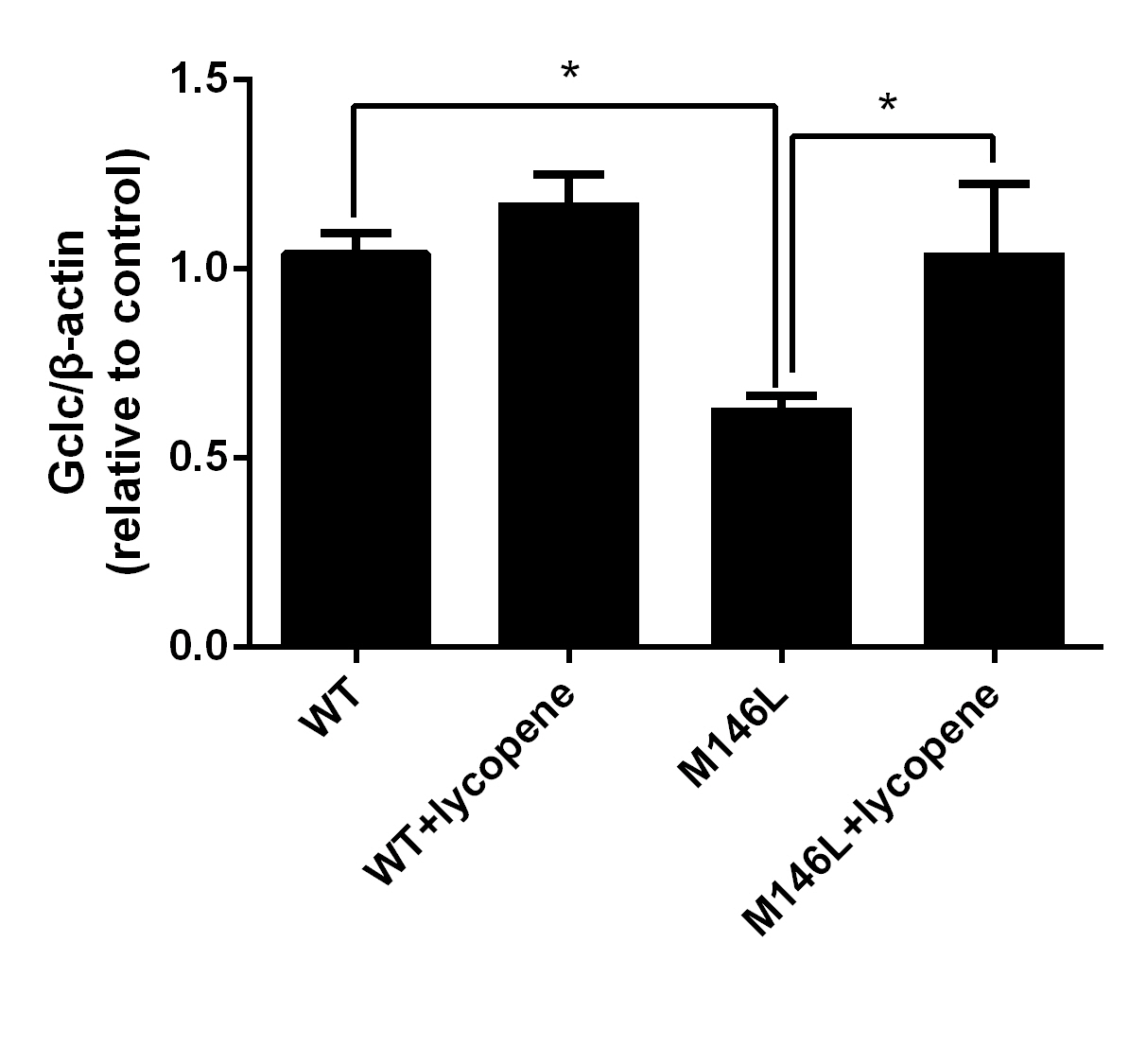

Supplement: Supplemental Information 1 [file peerj-08-9308-s001.zip › Raw date/GCLC-2.jpg]

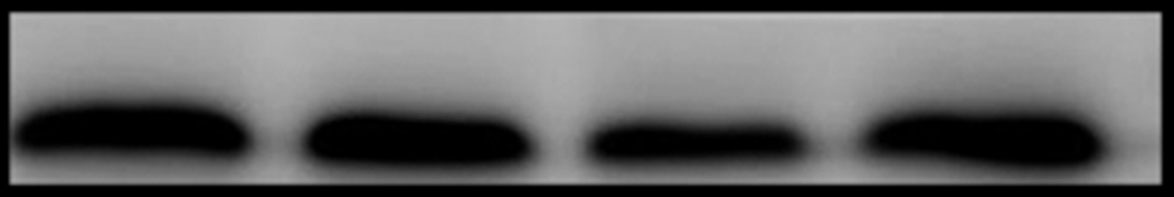

Supplement: Supplemental Information 1 [file peerj-08-9308-s001.zip › Raw date/gclc.jpg]

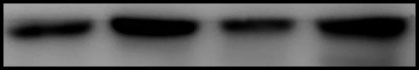

Supplement: Supplemental Information 1 [file peerj-08-9308-s001.zip › Raw date/gclm 2019.05.07.jpg]

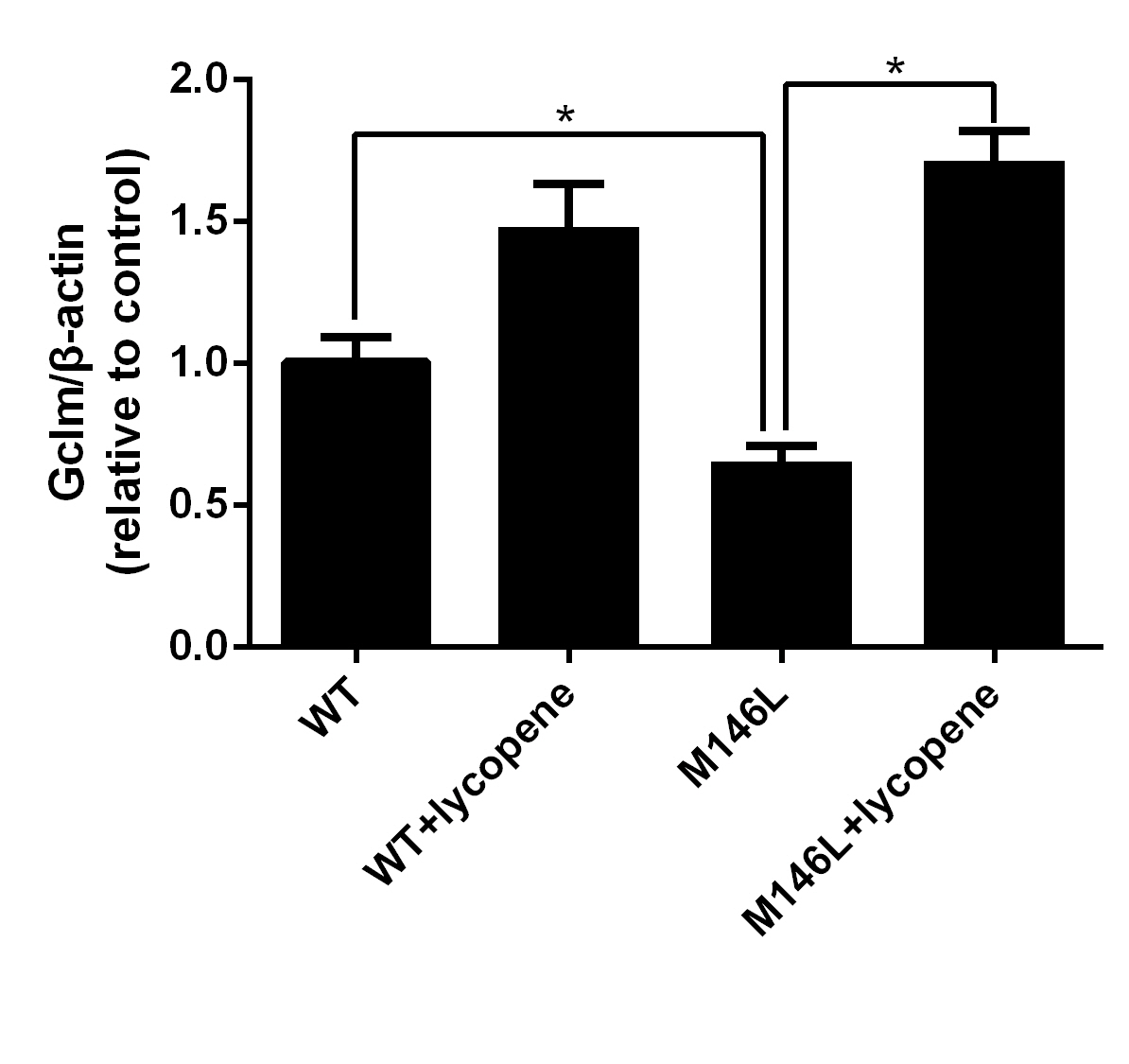

Supplement: Supplemental Information 1 [file peerj-08-9308-s001.zip › Raw date/GCLM-2.jpg]

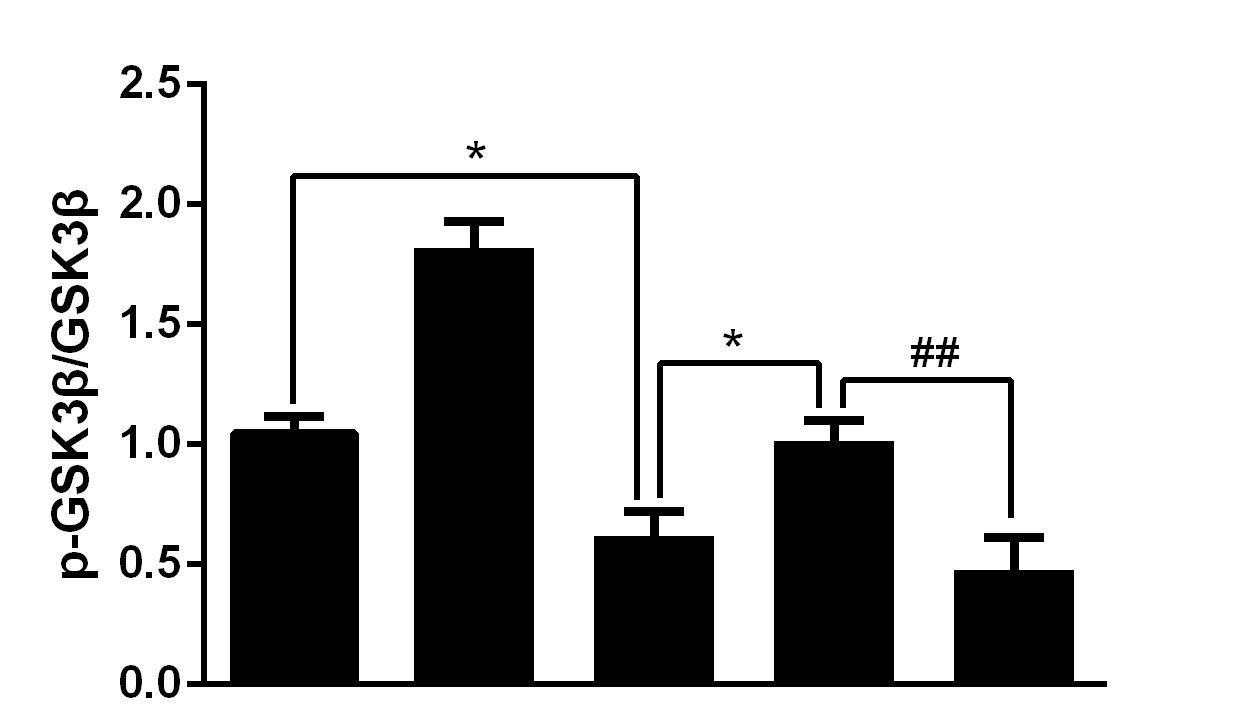

Supplement: Supplemental Information 1 [file peerj-08-9308-s001.zip › Raw date/GSK.jpg]

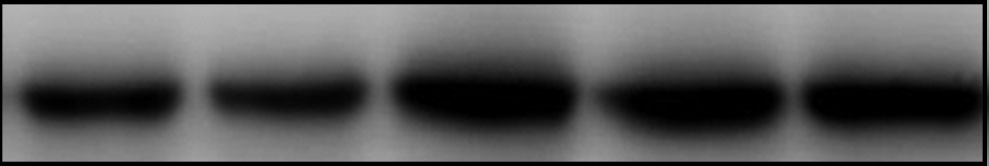

Supplement: Supplemental Information 1 [file peerj-08-9308-s001.zip › Raw date/GSK3B 2019.06.25.jpg]

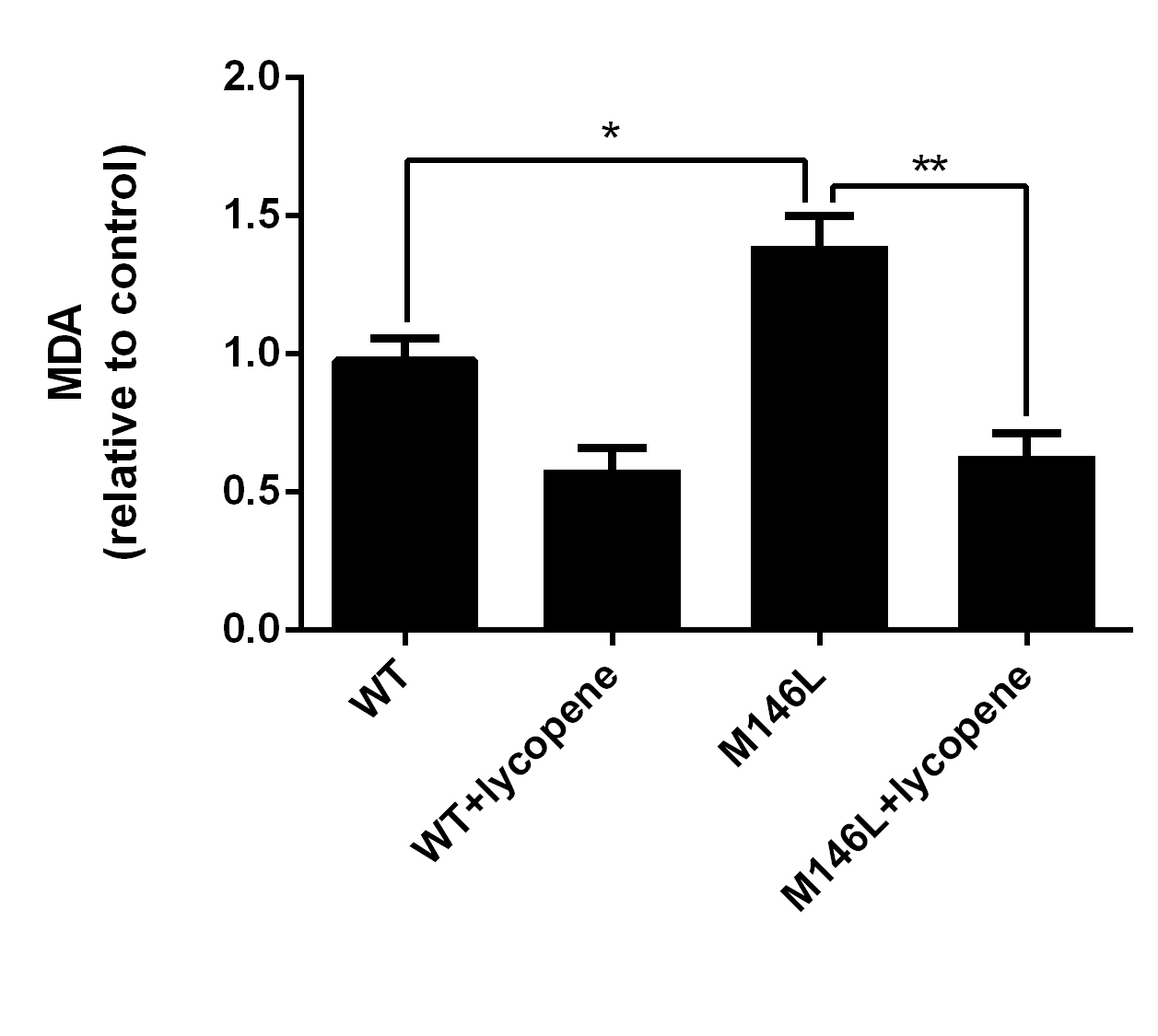

Supplement: Supplemental Information 1 [file peerj-08-9308-s001.zip › Raw date/MDA.jpg]

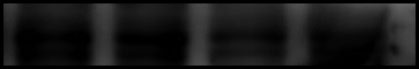

Supplement: Supplemental Information 1 [file peerj-08-9308-s001.zip › Raw date/nrf2 2019.05.27.jpg]

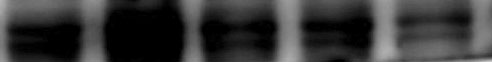

Supplement: Supplemental Information 1 [file peerj-08-9308-s001.zip › Raw date/nrf2 2019.05.27_11.07.19_Ch+Marker.jpg]

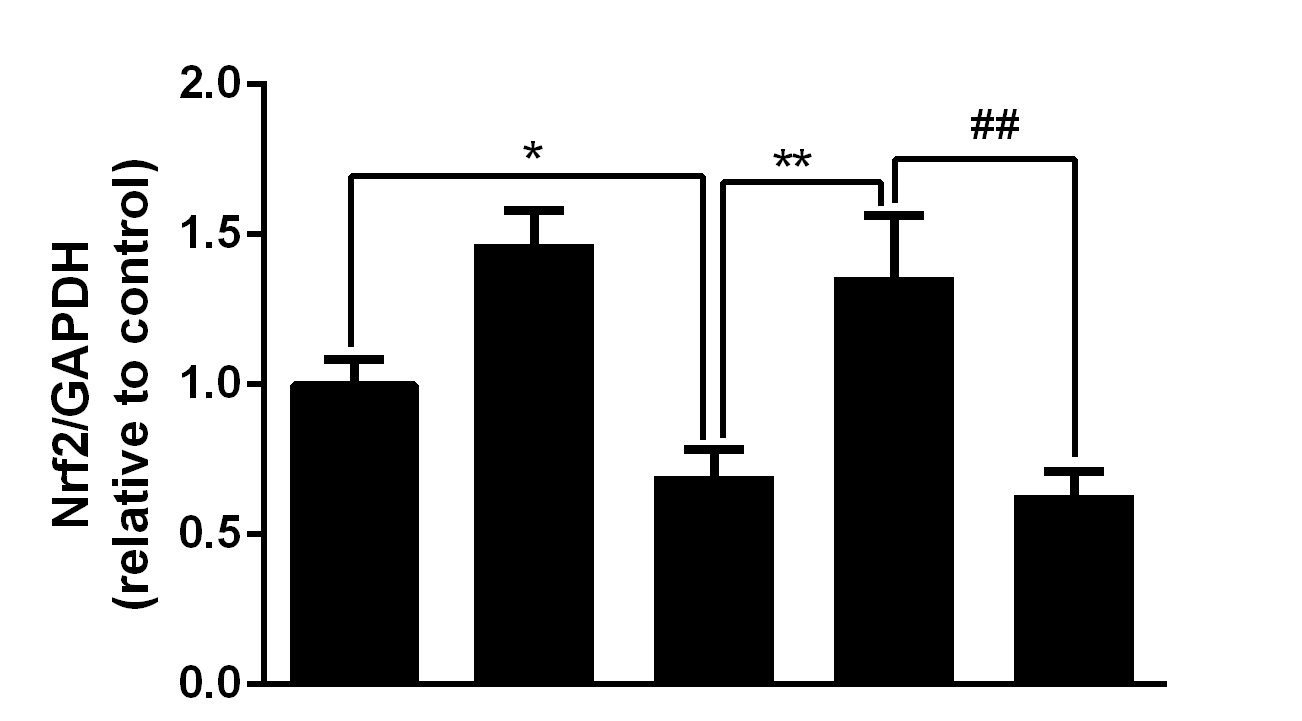

Supplement: Supplemental Information 1 [file peerj-08-9308-s001.zip › Raw date/NRF2-2.0.jpg]

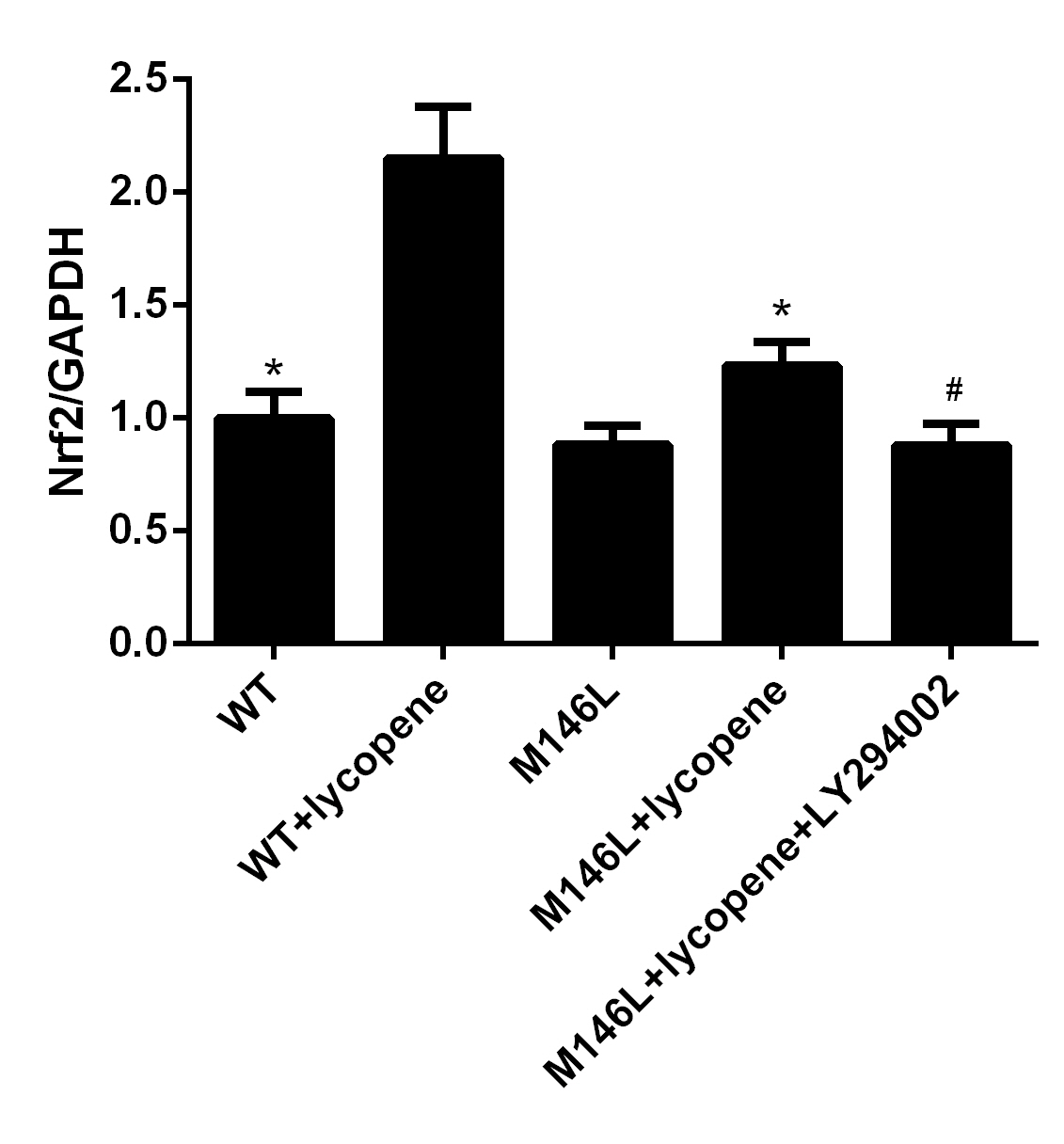

Supplement: Supplemental Information 1 [file peerj-08-9308-s001.zip › Raw date/NRF2.jpg]

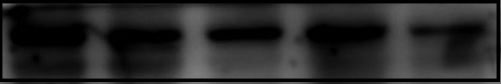

Supplement: Supplemental Information 1 [file peerj-08-9308-s001.zip › Raw date/p-AKT 2019.06.14.jpg]

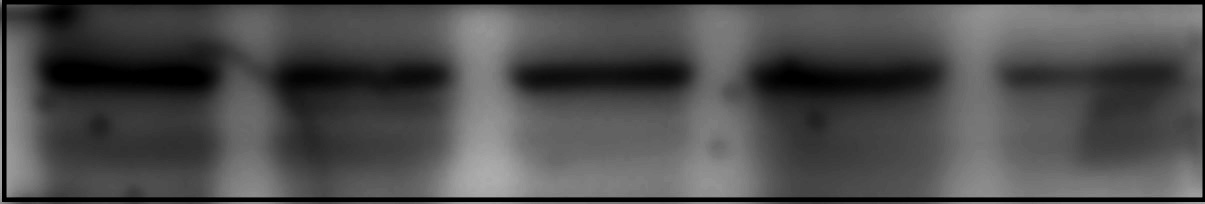

Supplement: Supplemental Information 1 [file peerj-08-9308-s001.zip › Raw date/p-AKT-3.jpg]

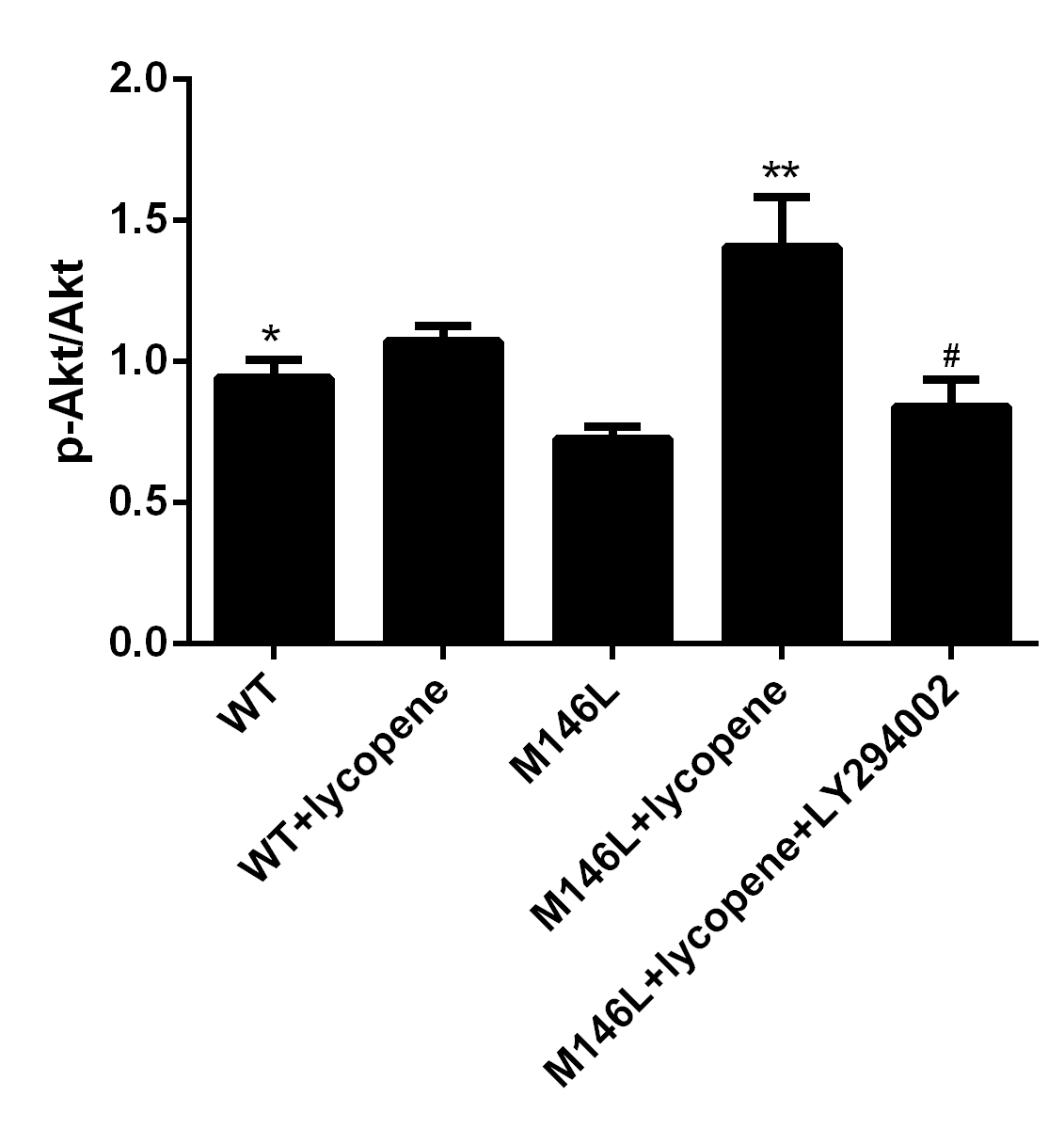

Supplement: Supplemental Information 1 [file peerj-08-9308-s001.zip › Raw date/p-AKT.jpg]

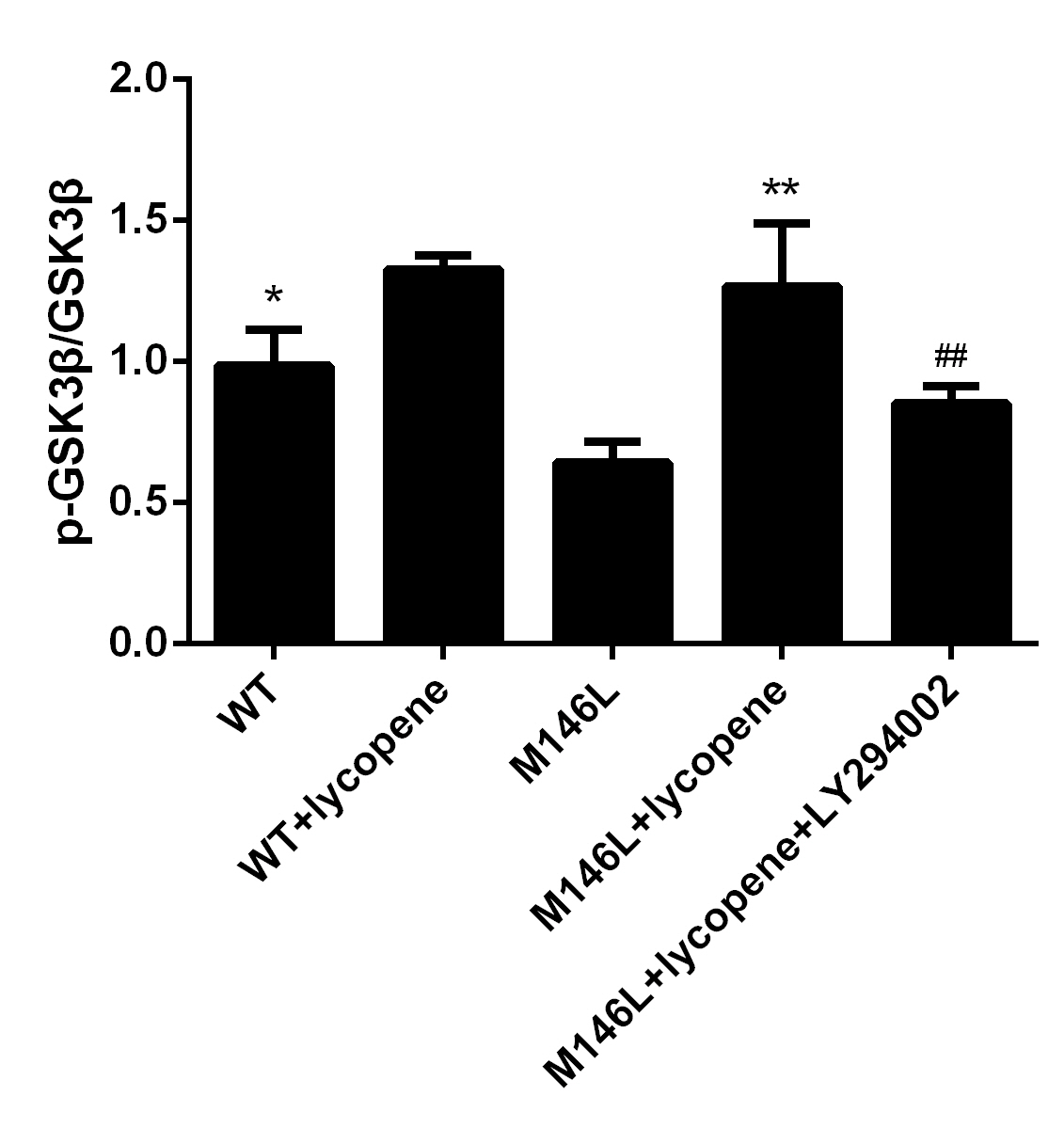

Supplement: Supplemental Information 1 [file peerj-08-9308-s001.zip › Raw date/p-GSK.jpg]

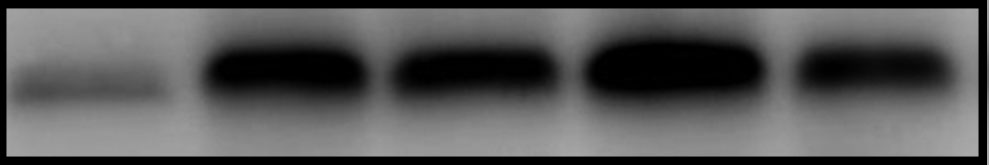

Supplement: Supplemental Information 1 [file peerj-08-9308-s001.zip › Raw date/p-GSK3B 2019.06.25.jpg]

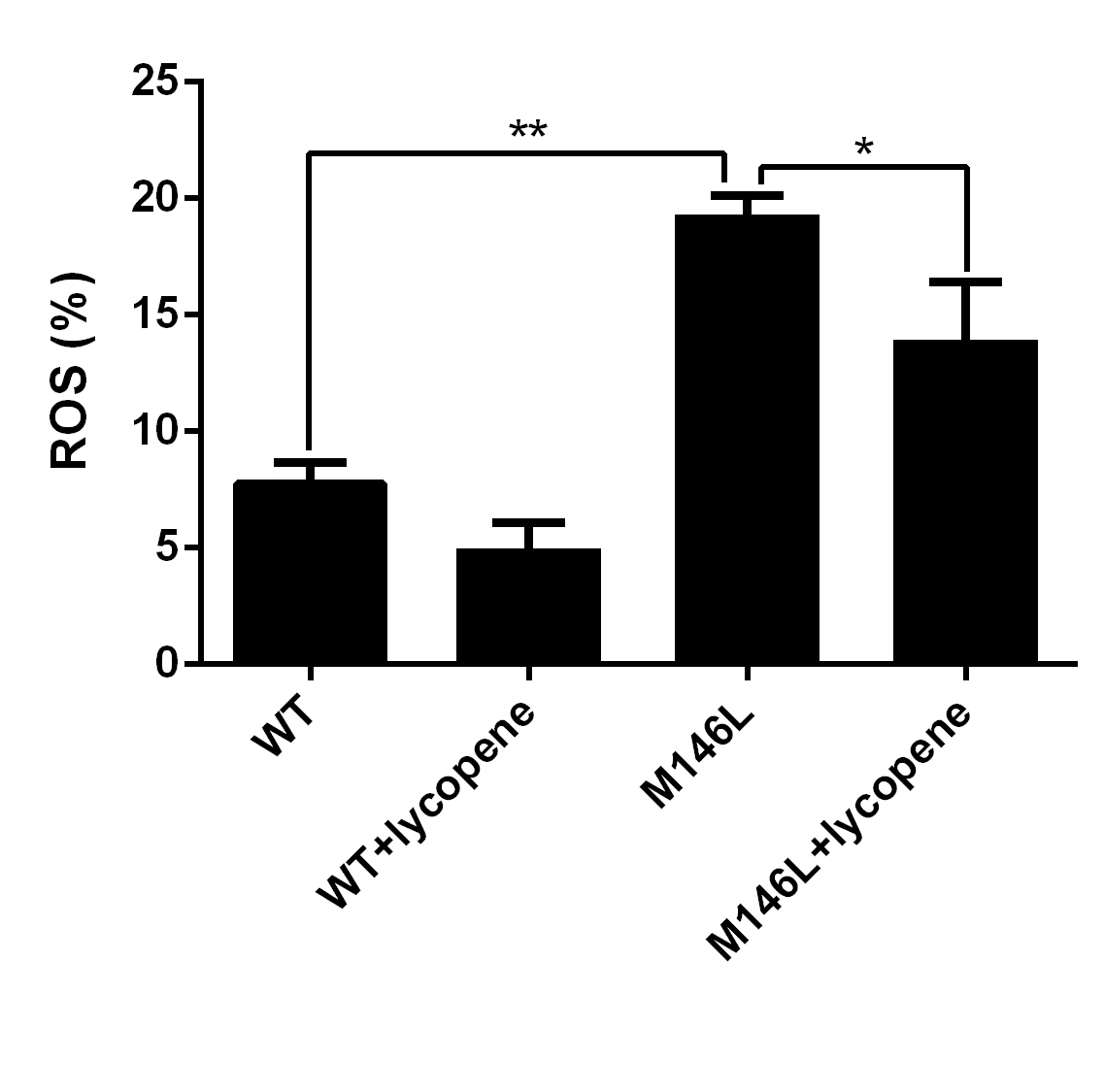

Supplement: Supplemental Information 1 [file peerj-08-9308-s001.zip › Raw date/ROS.jpg]
